# Evolutionary relationship between organellar DNA inheritance patterns and traits of seed plants

**DOI:** 10.1002/ajb2.70272

**Published:** 2026-10-11

**Authors:** Takuya Konuma, Takashi Makino

**Affiliations:** ^1^ Graduate School of Life Sciences Tohoku University 6‐3, Aramaki Aza Aoba, Aoba‐ku Sendai 980‐8578 Japan

**Keywords:** biparental inheritance, functional traits, paternal inheritance, phylogenetic comparative analysis, plastid inheritance

## Abstract

**Premise:**

In plants, organellar DNA is predominantly maternally inherited, but biparental and paternal inheritance have also been observed. Specific mechanisms, such as plastid exclusion during pollen mitosis or genome degradation, ensure maternal inheritance. Certain factors, including low temperatures, disrupt cytoskeletal functions, thereby increasing paternal plastid transmission. Despite these findings, the ecological and evolutionary drivers of these inheritance modes remain unclear. We explored the traits associated with biparental and paternal inheritance to reveal the contributing ecological factors.

**Methods:**

Plastid inheritance data were compiled for 378 species (302 genera and 112 families) and 12 functional traits obtained from the TRY database were analyzed. Missing values were filled according to Bayesian hierarchical modeling. On the basis of phylogenetic logistic regression, we investigated the relationships between inheritance patterns and plant traits, accounting for evolutionary history.

**Results:**

Our models identified plant height, leaf area, and stem conduit density as traits associated with inheritance modes, with the strongest support for leaf area and limited support for the other traits. Coefficient directions indicated greater plant height, smaller leaves, and higher stem conduit density in species with non‐maternal inheritance. Excluding gymnosperms yielded similar tendencies, which suggests that similar ecological factors may be associated with plastid inheritance patterns in both groups.

**Conclusions:**

Functional traits were associated with organelle inheritance patterns, with ecological and evolutionary implications. Similar ecological factors may be associated with the independent occurrence of paternal inheritance across lineages. Future work integrating molecular and ecological data will help elucidate the mechanisms underlying organelle inheritance and their adaptive significance.

From microorganisms to multicellular organisms, it is universally considered that organellar DNA is maternally inherited (Birky, 1995). The maternal inheritance of plastid (chloroplast) and mitochondrial DNA has been investigated across a wide range of species, including green algae such as *Volvox*, mosses, ferns, and seed plants, the latter being the most extensively studied (Gastony and Yatskievych, 1992; Guillon and Raquin, 2000; Natcheva and Cronberg, 2007; Kuroiwa, 2010; Wang et al., 2010; Kuo et al., 2018). Cytological studies showed that maternal inheritance is the predominant mode of inheritance in ~80% of angiosperms (Miyamura et al., 1987; Corriveau and Coleman, 1988, 1991; Zhang et al., 2003). Although maternal inheritance appears to be widespread among land plants, a number of exceptions—such as paternal and biparental inheritance—have been reported in seed plants. Examples include the biparental inheritance of plastids in *Medicago sativa*, which is in contrast to the maternal inheritance of mitochondria (Forsthoefel et al., 1992). Maternal inheritance of mitochondria and paternal inheritance of plastids have both been reported for *Actinidia deliciosa* (Testolin and Cipriani, 1997). Conversely, in *Musa acuminata*, plastids are maternally inherited, whereas mitochondria are paternally inherited (Fauré et al., 1994). These examples indicate that organelle inheritance in seed plants is not restricted to maternal inheritance, and it is clear that there are variable inheritance patterns (Mogensen, 1996). Furthermore, even in plant species in which maternal inheritance is considered predominant, exceptions have been detected. For example, in tobacco (*Nicotiana tabacum*) and arabidopsis (*Arabidopsis thaliana*), in which organelles are usually inherited maternally, plastid transmission through pollen has been observed, albeit at a very low frequency (Azhagiri and Maliga, 2007; Ruf et al., 2007). This phenomenon, known as “paternal leakage,” appears to be nearly universal when sufficiently large sample sizes are examined (Schneider, 2023; Sakamoto and Takami, 2024). These observations reflect the diversity in organelle inheritance.

Because of a cytological mechanism through which the organellar genome in seed plants is maternally inherited, plastids are often absent in male gametes during pollen development. In angiosperms, following meiosis, each microspore undergoes an asymmetric mitotic division (pollen mitosis I) to form one vegetative cell and one generative cell during the initial stage of microgametophyte development. The generative cell subsequently undergoes pollen mitosis II to form two sperm cells, which participate in fertilization. The following three mechanisms mediating the elimination of plastids have been confirmed in many plants, including tomato and corn: elimination during pollen mitosis, degradation in generative cells, and elimination during fertilization (Clauhs and Grun, 1977; Hagemann and Schröder, 1989; Hagemann, 2004). Recent mechanistic studies indicate that these processes act quantitatively rather than absolutely, with their relative contributions varying among species (Sakamoto and Takami, 2024). In addition, regarding plastid elimination during pollen mitosis I, plastid inheritance in plants is reportedly influenced by both environmental and genetic factors (Chung et al., 2023). Specifically, environmental factors (e.g., temperature) affect asymmetric cell division, and paternal plastids are present in generative cells under low‐temperature conditions. Chilling stress destabilizes the cytoskeleton during gametogenesis and alters cell division patterns during pollen mitosis I, resulting in the incomplete elimination of plastids. Additionally, the exonuclease DPD1 degrades the organellar genome during the pollen maturation process, thereby genetically inactivating plastids that were not eliminated during pollen mitosis I. When both of these mechanisms, namely plastid elimination and genome degradation, are abnormal, the proportion of biparental inheritance increases. Thus, plastid inheritance is likely a complex trait influenced by various factors.

The complexity of paternal organelle elimination is thought to be related to increasing oxidative damage. Allen (1996) proposed that, from a physiological perspective, paternal mitochondrial DNA is susceptible to oxidative damage due to high energy consumption, which contributes to its exclusion. Reactive oxygen species, which are produced by electron transfer reactions associated with respiration, damage sperm cell mitochondria, leading to the elimination of paternal organellar DNA during fertilization. Compared with paternal mitochondria, maternal mitochondria consume less energy and are less susceptible to oxidative damage. Moreover, the egg cell within the female gametophyte is protected by surrounding ovular tissues, and its relatively low metabolic activity may reduce oxidative damage to organellar DNA. In fact, higher organellar DNA mutation rates have been observed in species with paternal inheritance than in species with maternal inheritance (Whittle and Johnston, 2002; Drouin et al., 2008; Crosby and Smith, 2012). Although this hypothesis was originally developed in the context of mitochondrial inheritance, similar ideas have sometimes been speculated, by analogy, for plastid inheritance (Greiner et al., 2015). However, this fails to explain the various modes of inheritance that exist in addition to maternal inheritance.

Thus, there is considerable diversity in the mode of organellar DNA inheritance and the underlying mechanism, but even plants in the same genus (e.g., tomato and potato) differ in terms of the mechanisms mediating plastid elimination. More specifically, in tomato, male generative cells do not contain plastids, whereas in potato, paternal plastids are eliminated late during gametogenesis (Bock, 2007), suggesting that organelle inheritance patterns evolved independently in multiple lineages (Greiner et al., 2015). These previous studies suggest that there are ecological factors related to the mode of organellar DNA inheritance, but no studies have linked traits to the mode of inheritance in plant species. Furthermore, it is unclear how these factors affect the mode of inheritance. Therefore, the objectives of this study were to identify traits common among species with biparental or paternal inheritance and to reveal the contributing ecological factors.

## MATERIALS AND METHODS

### Data collection

On the basis of several published articles, we obtained the genetic patterns of the plastid genomes from 378 species (302 genera and 112 families; Table 1, Figure 1, and Appendix S1). Taxonomic authorities were obtained from the GBIF taxonomic backbone and verified or supplemented using *Plants of the World Online* (POWO) (GBIF Secretariat, 2023; POWO, 2026). In most studies, the mode of plastid genome inheritance was classified into three patterns: maternal, biparental, and paternal. In this study, by contrast, we distinguished between maternal (0) and non‐maternal (1) inheritance for our analysis. Next, we obtained data for 18 traits (Table 2) reported by Bruelheide et al. (2018) as functional traits affecting different key ecosystem processes and likely responsive to macroclimatic drivers for all species in the TRY database version 5.0 (accessed on November 17, 2021; Kattge et al., 2020). We used the mean of individual sample values from these data as the species‐level value for each target trait (Appendix S2). The obtained values were log_10_‐transformed and standardized for a distribution with zero mean and unit variance (*z*‐transformation). We considered traits for which values were obtained in >5% of all species. We also excluded species that had no values for any of these traits. Accordingly, we obtained data containing gaps for 12 traits: leaf area (LA), leaf dry mass per area (LMA), leaf dry matter content (LDMC), leaf carbon (LeafC), nitrogen (LeafN) and phosphorus (LeafP) contents per leaf dry mass, leaf nitrogen content per leaf area (LNA), leaf N/P ratio (LNP), seed dry mass (SM), plant height (H), stem dry mass per stem fresh volume (SSD), and stem conduit density (SCD) of 45,400 species. To improve the accuracy of gap filling, we first performed imputation on 45,400 species using Bayesian hierarchical probabilistic matrix factorization (Shan et al., 2012; Schrodt et al., 2015) and then assigned trait values to species for which the mode of inheritance was known (Appendix S3). For a logistic regression analysis considering phylogenetic relationships, we created a genus‐level phylogenetic tree by pruning the species‐level plant phylogeny of Zanne et al. (2014) such that terminal nodes corresponded to genera. Among the 378 species analyzed, 322 species were present in the phylogeny of Zanne et al. (2014), whereas all genera were represented. Following the approach of Tateyama et al. (2021), we randomly selected one available species per genus to avoid phylogenetic pseudoreplication. The remaining species within each genus were subsequently incorporated by assigning the same phylogenetic distance (0.000001) within a genus, thereby approximating zero divergence among congeneric species. A total of 378 species were included in the resulting tree.

**Table 1:** List of 112 families used for the phyloglm models and the number of species exhibiting plastid inheritance patterns.

	*n*			*n*			*n*	
Family	M	P/B		M	P/B		M	P/B
Acanthaceae	2	0	Cupressaceae	1	6	Phrymaceae	1	0
Actinidiaceae	0	1	Cyperaceae	0	1	Phytolaccaceae	2	0
Aizoaceae	1	1	Dioscoreaceae	1	0	Pinaceae	0	11
Amaranthaceae	2	0	Droseraceae	0	2	Pittosporaceae	1	0
Apiaceae	3	0	Ebenaceae	1	0	Plantaginaceae	3	0
Apocynaceae	7	0	Ericaceae	0	1	Plumbaginaceae	1	3
Araceae	1	1	Eucommiaceae	1	0	Poaceae	17	1
Araliaceae	1	0	Euphorbiaceae	6	0	Polemoniaceae	2	0
Araucariaceae	0	1	Fabaceae	38	15	Polygalaceae	3	0
Arecaceae	2	0	Gentianaceae	3	0	Polygonaceae	3	0
Aristolochiaceae	1	0	Geraniaceae	0	4	Pontederiaceae	1	0
Asclepiadaceae	2	0	Gesneriaceae	1	0	Portulacaceae	1	1
Asparagaceae	1	0	Hamamelidaceae	2	0	Primulaceae	2	0
Asteraceae	11	0	Hippocastanaceae	1	0	Ranunculaceae	9	0
Balsaminaceae	1	1	Hydrangeaceae	1	0	Rosaceae	12	0
Berberidaceae	1	0	Hydrophyllaceae	1	0	Rubiaceae	6	0
Bignoniaceae	3	0	Iridaceae	3	0	Rutaceae	2	0
Bixaceae	1	0	Juglandaceae	1	0	Salicaceae	2	0
Boraginaceae	2	1	Lamiaceae	6	0	Sapindaceae	2	0
Brassicaceae	6	0	Liliaceae	6	1	Saxifragaceae	2	0
Bromeliaceae	1	0	Linaceae	0	1	Scrophulariaceae	8	0
Buxaceae	1	0	Loganiaceae	1	0	Simaroubaceae	1	0
Cactaceae	4	1	Lythraceae	3	0	Solanaceae	15	0
Calycanthaceae	1	0	Magnoliaceae	1	0	Sterculiaceae	2	0
Campanulaceae	0	5	Malpighiaceae	1	0	Strelitziaceae	0	1
Cannabaceae	1	0	Malvaceae	3	2	Theaceae	1	0
Cannaceae	1	0	Moraceae	4	0	Tropaeolaceae	1	0
Capparaceae	1	0	Musaceae	1	0	Turneraceae	0	1
Caprifoliaceae	1	10	Myrtaceae	2	0	Typhaceae	1	0
Caryophyllaceae	4	1	Nyctaginaceae	1	0	Ulmaceae	1	0
Cephalotaxaceae	0	1	Nymphaeaceae	1	0	Urticaceae	1	0
Chenopodiaceae	2	0	Oleaceae	5	2	Verbenaceae	5	0
Clusiaceae	0	2	Onagraceae	1	5	Violaceae	1	0
Commelinaceae	2	0	Orchidaceae	2	0	Vitaceae	1	0
Convolvulaceae	0	1	Oxalidaceae	2	0	Zingiberaceae	1	0
Cornaceae	2	1	Papaveraceae	4	0	Zygophyllaceae	1	0
Crassulaceae	3	0	Passifloraceae	0	1			
Cucurbitaceae	1	0	Pedaliaceae	1	0			

*Note*: M = maternal; P/B = paternal/biparental.

**Figure 1:**
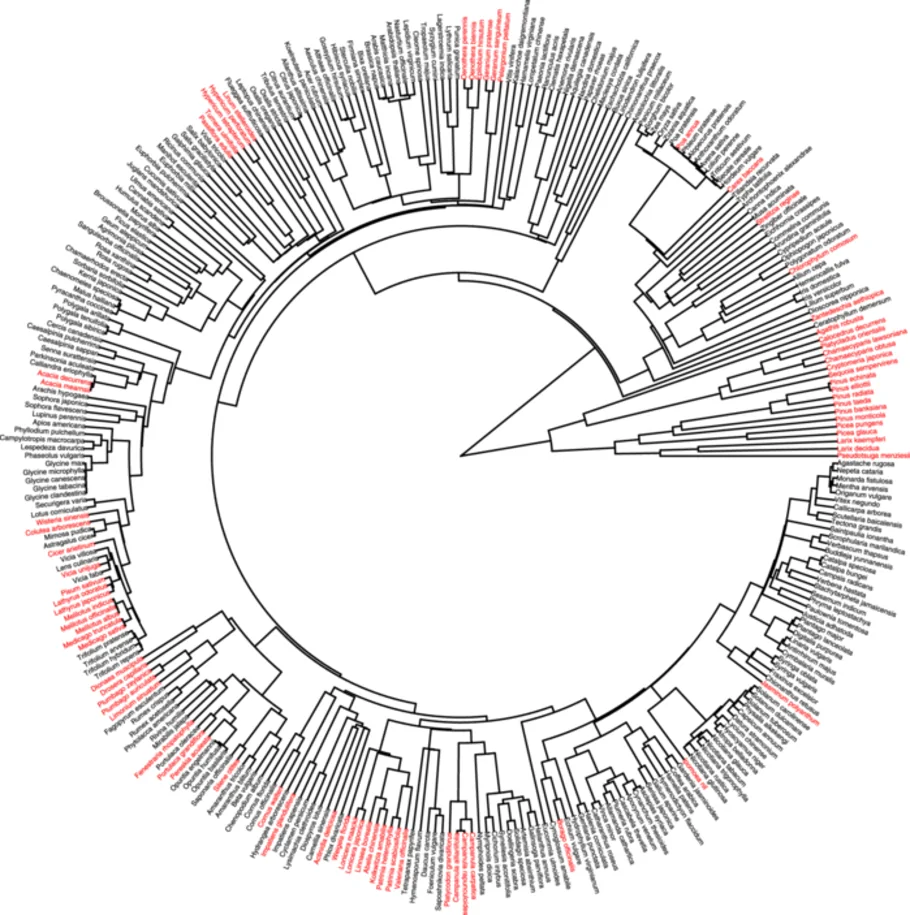
Phylogenetic tree used for the phyloglm models. Of the 378 species analyzed by phyloglm, 322 species were present in the phylogeny of Zanne et al. (2014) (246 of 291 maternal species and 76 of 87 non‐maternal species). The remaining 56 species were incorporated into the phylogeny assuming the same phylogenetic distance (0.000001) within a genus because all genera were represented in the phylogeny. The names of the non‐maternal species are highlighted in red.

**Table 2:** Eighteen functional traits used for this research.

Trait	Abbreviation	Unit
Leaf area	LA	mm^2^
Leaf dry mass per leaf area	LMA	g/m^2^
Leaf fresh mass	LFM	g
Leaf dry matter content (i.e., leaf dry mass per leaf fresh mass)	LDMC	g/g
Leaf carbon (C) content per leaf dry mass	LeafC	g/g
Leaf nitrogen (N) content per leaf dry mass	LeafN	g/g
Leaf phosphorus (P) content per leaf dry mass	LeafP	g/g
Leaf nitrogen (N) content per leaf area	LNA	g/m^2^
Leaf nitrogen/phosphorus (N/P) ratio	LNP	g/g
Leaf nitrogen isotope ratio	d15N	per mil
Seed dry mass	SM	mg
Seed length	SL	mm
Seed number per reproductive unit	SNRU	*n*
Dispersal unit length	DL	mm
Plant height	H	m
Stem specific density (i.e., stem dry mass per stem fresh volume)	SSD	g/cm^3^
Stem conduit density	SCD	*n*/mm^2^
Conduit element length	CEL	μm

### Data analysis

To predict the relationship between the mode of plastid genome inheritance and functional traits, we constructed a phylogenetic generalized linear model using the mode and traits as objective and explanatory variables, respectively. The R phyloglm function from the package phylolm was used for the analysis (Tung Ho and Ané, 2014). This approach allowed us to fit phylogenetic generalized linear models to the traits while accounting for the expected covariance due to shared evolutionary history. The Akaike information criterion (AIC) was used to select the best‐fitting model. This criterion evaluates the trade‐off between model fit and complexity, allowing us to identify the most parsimonious model that adequately explains the data. Because the phylogenetic tree used in this method was created by a partially random process, the variable selection results were unstable. To solve this problem, the AIC values of all combinations of variables were obtained for the 100 phylogenetic trees created using this method (i.e., 100 AIC values for one model). The lowest median value was used to determine the best model. For each candidate model, ΔAIC was calculated as the difference between its median AIC and the lowest median AIC. Akaike weights were calculated from these ΔAIC values across the full set of candidate models. In addition, 20 of the 378 species were conifers with paternal or biparental inheritance patterns, which may lead to a bias for traits. Therefore, we performed the same analysis for non‐coniferous samples.

To evaluate the robustness of the detected associations and assess their sensitivity to taxonomically structured trait imputation and particular phylogenetic clades, we first summarized the numbers and proportions of observed and imputed values for each trait included in the analyses. We then performed a leave‐one‐clade‐out (LOCO) sensitivity analysis. Specifically, we sequentially excluded eight families containing relatively large numbers (≥3) of non‐maternal species (Fabaceae, Pinaceae, Caprifoliaceae, Cupressaceae, Onagraceae, Campanulaceae, Geraniaceae, and Plumbaginaceae) and repeated the entire analytical procedure, including phylogenetic tree construction and exhaustive model selection across all combinations of explanatory variables. As in the primary analysis, 100 phylogenetic trees were generated for each data set, and the median AIC across replicates was used to identify the best‐supported model. To account for uncertainty in selecting a single best‐supported model, we also calculated the ΔAIC between the best‐supported and runner‐up models and the Akaike weight of the best‐supported model across the full set of candidate models for each LOCO analysis. We then summarized how frequently each trait included in the original best‐supported model was retained in the best‐supported models across the LOCO analyses to evaluate the consistency of the identified trait associations across clade exclusions, while taking model‐selection uncertainty into account.

Finally, we assessed potential effects of trait imputation using only observed trait values. Because only 13 species, including three non‐maternal species, had observed values for H, LA, and SCD, SCD could not be evaluated reliably in the observed‐only analysis. We therefore restricted model selection to the 131 species with observed values for both H and LA and compared four candidate models: the null, H‐only, LA‐only, and H + LA models. As in the primary analysis, models were fitted across 100 phylogenetic trees, and the median AIC across replicates was used for model comparison. ΔAIC and Akaike weights were calculated from the median AIC values as described above.

## RESULTS

Using the generated data of 12 functional traits and plastid inheritance modes, biparental and paternal inheritance were known in 87 of 378 species. Figure 2 shows the distributions of the 12 gap‐filled trait values according to plastid inheritance mode. Using these traits, we created 4095 combinations of variables, built 100 models for each, and calculated the median AIC value for each combination. The AIC values of the five models with the lowest median values are shown in Figure 3. The single lowest‐AIC model included H, LA, LNP, and SCD (median AIC = 270.26; Table 3 and Figure 3A). However, the five best‐supported models had ΔAIC values ranging from zero to 1.35 and Akaike weights ranging from 0.02 to 0.03, indicating that these models could not be clearly distinguished on the basis of AIC. In the single lowest‐AIC model, the median coefficients across the 100 phylogenetic trees were positive for H (0.254) and SCD (0.158) and negative for LA (−0.392) and LNP (−0.115) (Figure 4A). Similarly, when conifers were excluded from our analyses, the best model included H, LA, LeafN, LNA, and SCD (median AIC value = 262.22; Figure 3B), with median coefficients of 0.204, −0.433, −0.131, −0.093, and 0.110, respectively (Figure 4B).

**Figure 2:**
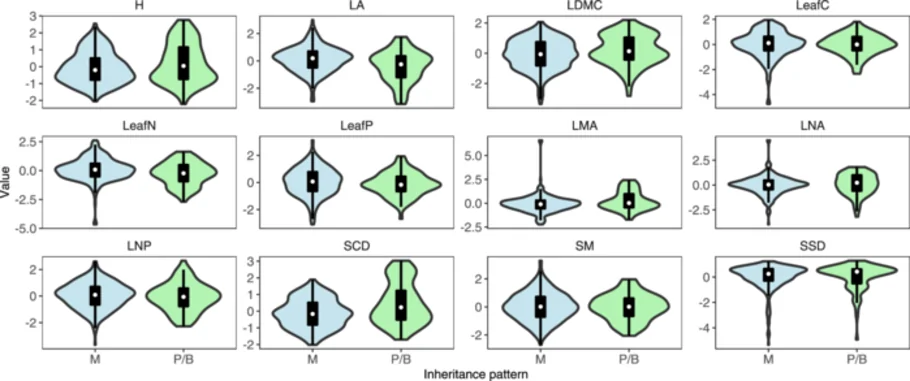
Comparison of 12 traits used in the model in relation to inheritance patterns (for trait abbreviations, see Table 2). Distribution of trait values for 291 species with maternal inheritance (M, blue) and 87 species with paternal/biparental inheritance (P/B, green). Values are log_10_‐transformed and standardized for a distribution with zero mean and unit variance (*z*‐transformation). White dot = median; thick black line = interquartile range; thin black line = 1.5× interquartile range.

**Figure 3:**
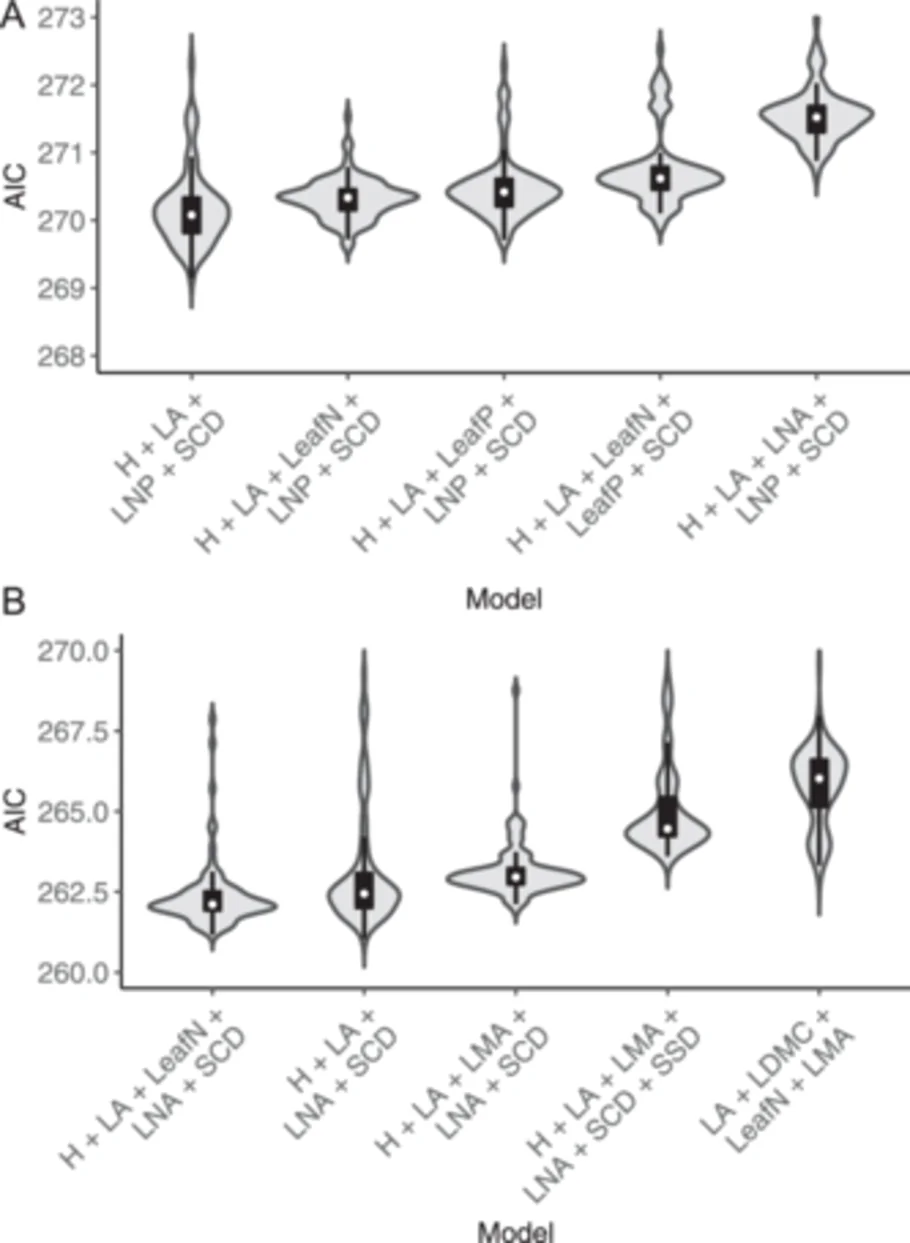
AIC values for the five phyloglm models with the best fit. AIC values were calculated for each of 4095 models using all combinations of 12 traits for (A) all 378 samples and (B) 358 samples (i.e., excluding 20 conifer species) across 100 phylogenetic trees. The distribution for the five models with the lowest median values is presented. White dot = median; thick black line = interquartile range; thin black line = 1.5× interquartile range. To facilitate comparison of medians, the range for A is displayed between 268 and 273, and that for B between 260 and 270.

**Table 3:** Model‐selection results for the five best‐supported models, with Akaike weights based on all 4095 candidate models.

Model	Median AIC	ΔAIC	Akaike weight
H + LA + LNP + SCD	270.26	0.00	0.03
H + LA + LeafN + LNP + SCD	270.34	0.09	0.03
H + LA + LeafP + LNP + SCD	270.44	0.18	0.03
H + LA + LeafN + LeafP + SCD	270.67	0.42	0.02
H + LA + LNA + LNP + SCD	271.61	1.35	0.02

**Figure 4:**
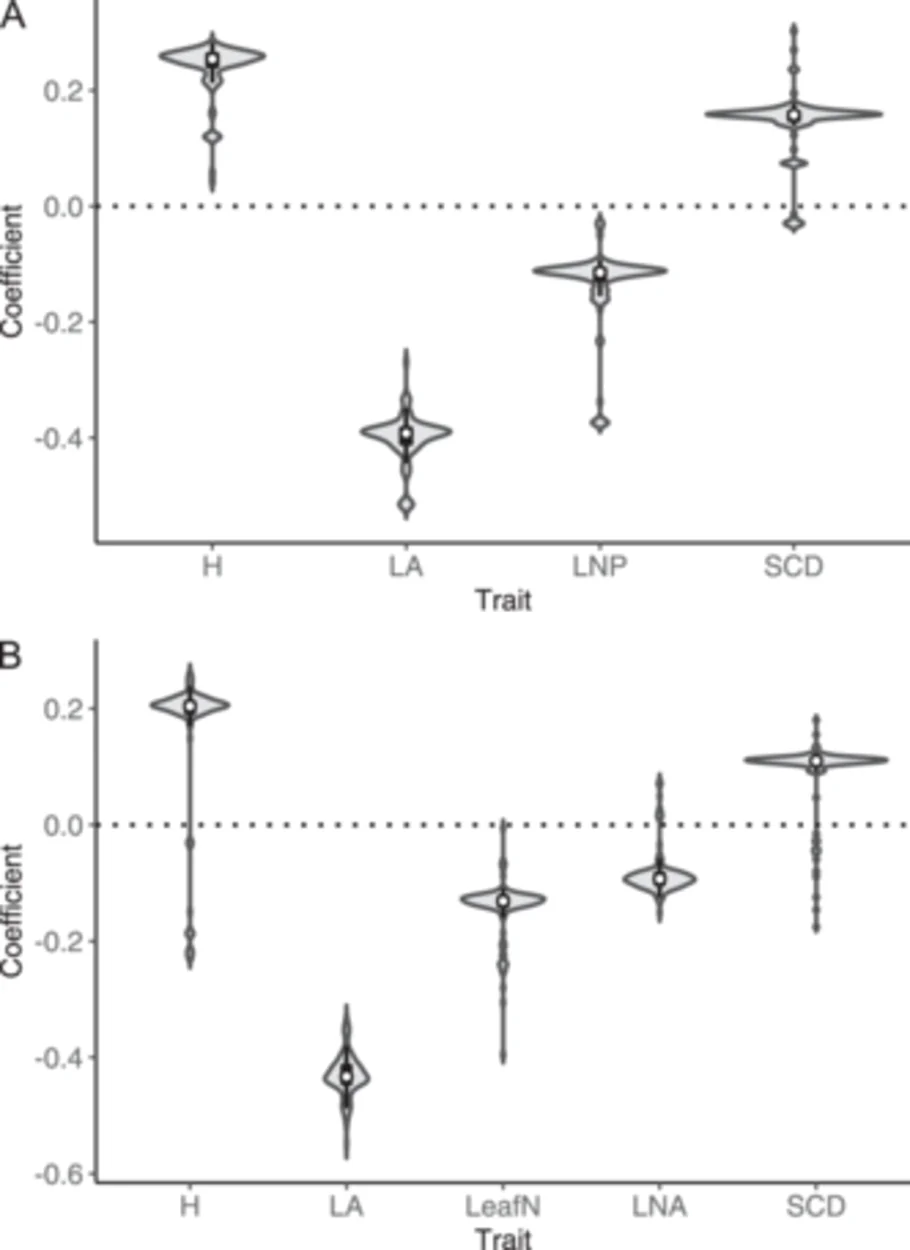
Coefficients of the model with the best fit. Coefficients were calculated for the model with the lowest AIC value for (A) all 378 samples and (B) 358 samples (i.e., excluding 20 conifer species) across 100 phylogenetic trees. White dot = median; thick black line = interquartile range; thin black line = 1.5× interquartile range.

The proportions of observed and imputed values varied considerably among traits (Appendix S4). Across the eight LOCO analyses, LA was retained in the best‐supported model in all analyses (8/8), whereas H and SCD were retained in six analyses each. By contrast, LNP was not retained in any of the best‐supported models (Appendix S5). Other leaf traits were not retained consistently across the LOCO analyses. Across all eight LOCO analyses, the ΔAIC between the best‐supported and runner‐up models was <1 (range: 0.01–0.72), and the Akaike weights of the best‐supported models were low (0.013–0.022; Appendix S5). Among the 378 species, empirical observations were available for 304 species for H, 133 for LA, and only 34 for SCD (Appendix S4). Among the 87 species with non‐maternal inheritance, only six had empirically observed SCD values. Furthermore, no empirical SCD values were available for species in five of the eight families examined in the LOCO analyses (Pinaceae, Campanulaceae, Geraniaceae, Onagraceae, and Plumbaginaceae).

We then examined associations involving H, LA, and SCD using only observed trait values. Because only 13 species, including three non‐maternal species, had observed values for all three traits, SCD could not be evaluated reliably. We therefore restricted the observed‐only model selection to the 131 species with observed values for both H and LA (Table 4). Model selection identified the LA‐only model as the best‐supported model (median AIC = 109.36, ΔAIC = 0, Akaike weight = 0.69), followed by the H + LA model (median AIC = 110.92, ΔAIC = 1.56, Akaike weight = 0.31). The H‐only and null models had substantially higher median AIC values (132.29 and 206.15, respectively; ΔAIC = 22.93 and 96.80; Akaike weights <0.01).

**Table 4:** Model‐selection results based on 131 species with empirically observed values for both H and LA.

Model	Coefficient		Median AIC	ΔAIC	Akaike weight
	H	LA			
LA	–	−0.35	109.36	0.00	0.69
H + LA	0.09	−0.38	110.92	1.56	0.31
H	0.37	–	132.29	22.93	0.00
Null	–	–	206.15	96.80	0.00

## DISCUSSION

### Traits associated with the ecological strategy considered to be relevant to genetic inheritance

In the best‐supported model based on AIC, plant height (H), leaf area (LA), leaf nitrogen/phosphorus (N/P) ratio (LNP), and stem conduit density (SCD) were initially selected as traits associated with the mode of plastid genome inheritance. However, the LOCO analyses indicated that the association involving LNP was not robust, whereas the associations involving H, LA, and SCD were retained consistently or in most analyses. Therefore, the following discussion focuses on these three traits, with the strongest support for LA and more limited support for H and SCD. Plant height is a crucial factor influencing plant ecological strategies. Height is a major determinant of the ability of a plant to compete for light, while also being a central component affecting carbon acquisition; a previous study revealed the correlation between H and various traits, including leaf mass fraction, leaf area ratio, leaf nitrogen per area, leaf mass per area, and canopy area (Falster and Westoby, 2003). Additionally, H has important regulatory effects on several life‐history traits, including seed abundance, time to reproduction, lifespan, and annual seed production (Moles and Leishman, 2008). The correlation between H and various traits suggests that plastid inheritance is determined by complex ecological factors as well as complex cytological mechanisms. Although no previous studies have revealed an association between H and paternal inheritance, H might indirectly affect inheritance through correlated ecological traits.

Leaf area tends to increase in warm and humid environments because of improved photosynthetic efficiency. Conversely, in cold and dry environments, leaf size decreases, which is conducive to decreasing water loss and mitigating the detrimental effects of freezing stress. In plants, changes to LA are important for the efficient use of resources and the optimization of survival strategies (Wright et al., 2017). Our results indicate that decreases in LA are associated with higher frequencies of paternal or biparental inheritance, which is consistent with the findings of an earlier study by Chung et al. (2023), which determined that chilling stress significantly increases paternal plastid transmission, suggestive of evolutionary significance. However, whether this reflects a causal relationship remains unclear.

Stem conduit density is an important anatomical trait reflecting xylem structure and is closely associated with plant hydraulic strategies, including water transport efficiency and resistance to embolism (Hacke et al., 2006; Sperry et al., 2008). Higher conduit densities are often linked to hydraulic safety and are characteristic of species adapted to environments with fluctuating or stressful water conditions, while also covarying with growth rate, mechanical support, and whole‐plant resource allocation (Poorter et al., 2010; Wright et al., 2010). In our analysis, SCD showed a positive coefficient in the primary analysis and was retained in six of the eight LOCO analyses, although this association remains tentative because of the limited number of observed values. This tentative association raises the possibility that species with particular vascular and hydraulic strategies may be more prone to non‐maternal plastid transmission. Although vascular anatomy is not directly involved in plastid inheritance at the cellular level, SCD is known to correlate with suites of ecological and life‐history traits, including growth form and stress tolerance. Therefore, the observed association may reflect broader ecological syndromes rather than a direct causal relationship. Taken together, these results imply that plastid inheritance patterns may be shaped by integrated ecological and anatomical strategies, rather than by isolated physiological traits.

### Common traits affecting paternal inheritance in angiosperms and conifers

Paternal inheritance has been detected in angiosperms, but it also occurs in many coniferous species, which are gymnosperms. In conifers, plastids delivered by pollen enter the egg, whereas maternal plastids are excluded from the developing proembryo or subsequently degenerate, depending on the lineage, resulting in predominantly paternal plastid inheritance (Neale et al., 1989; Mogensen, 1996). In our statistical analyses, paternal and biparental inheritance were treated together as non‐maternal inheritance; however, the following discussion focuses qualitatively on paternal inheritance as a representative case, because conifers predominantly exhibit paternal plastid inheritance. Our analysis produced the same results when conifers were excluded, suggesting that the characteristic traits of species exhibiting non‐maternal inheritance are similar in conifers and angiosperms. Therefore, in terms of the evolutionary significance of the mode of plastid inheritance, it is notable that species exhibiting paternal or biparental inheritance with the same trait tendencies as the typical species exhibiting paternal inheritance (i.e., conifers) appear multiple times independently in the phylogenetic tree. Although our results support the above‐mentioned findings, further molecular‐biology‐based or population‐genetic experiments and analyses are needed to elucidate the precise mechanisms that restrict the dominance of maternal inheritance related to oxidative damage (Allen, 1996).

### Robustness of the identified trait associations

The LOCO analyses showed that LA was retained in the best‐supported model in all analyses, whereas H and SCD were retained in six of the eight analyses. Although the small ΔAIC values and low Akaike weights indicated some uncertainty in model selection, the repeated retention of these traits across the LOCO analyses suggests that the associations involving LA, and to a lesser extent H and SCD, were relatively consistent across clade exclusions. By contrast, LNP was not retained in the best‐supported model in any LOCO analysis, indicating that its association with plastid inheritance was more sensitive to phylogenetic clade exclusion than those of LA, H, and SCD. The observed‐data analysis further supported the association involving LA: when model selection was restricted to empirically observed values of H and LA, the LA‐only model received the greatest support, followed by the H + LA model. Thus, the association involving LA was consistently recovered both across clade exclusions and in the analysis without imputed trait values.

### Study limitations and future challenges

This study has three main limitations related to the definition of inheritance mode, gap filling, and experimental validation.

First, there is ambiguity in how the objective variable is defined for logistic regression analyses. In this study, maternal inheritance (0) and non‐maternal inheritance (1) were used, but there are still problems with this binary classification. In addition to maternal inheritance, other inheritance patterns have been identified for organellar DNA, including paternal inheritance, biparental inheritance, and paternal leakage that occur at various rates. We are therefore concerned that a simple binary classification may obscure information about the diversity and frequency of non‐maternal inheritance patterns. To adequately reflect inheritance patterns, more detailed paternal leakage rates and additional data for species exhibiting paternal inheritance are needed. In addition, only 24 of the 87 non‐maternal species included in this study have been genetically confirmed, whereas the remaining species were classified on the basis of cytological evidence indicating the potential for biparental transmission rather than direct genetic demonstration of organellar inheritance. Although cytological observations provide valuable information for comparative analyses, this limitation should be considered when interpreting the inheritance categories. As additional genetically confirmed cases become available, the robustness of the associations identified here should be reevaluated.

Second, although the LOCO analyses showed relatively consistent retention of LA, H, and SCD across clade exclusions, the small ΔAIC values and low Akaike weights indicate uncertainty in the selection of a single best‐supported model. In addition, uncertainty associated with imputed trait values cannot be completely excluded, particularly for SCD, for which 91% of the trait values were obtained through BHPMF‐based imputation (Appendix S4). This limitation is particularly relevant for SCD because only 34 species had empirically observed values, including only six of the 87 species with non‐maternal inheritance, and no empirical SCD values were available for five of the eight families examined in the LOCO analyses. Consequently, SCD could not be evaluated in the observed‐data analysis. By contrast, the observed‐data analysis recovered LA in both of the two best‐supported models, with the LA‐only model receiving the greatest support, suggesting that the association involving LA was not solely attributable to trait imputation. H was included in the second‐best model but not in the best‐supported model, suggesting that its association should be interpreted more cautiously than that of LA. The association involving LNP was not consistently retained across the LOCO analyses (Appendix S5) and should therefore be interpreted with particular caution. Future studies incorporating more comprehensive empirical trait measurements, particularly for SCD, together with comparisons of alternative gap‐filling approaches, will help further validate these findings.

Finally, it is necessary to verify the results predicted by the model. In this study, we constructed a model on the basis of the mode of organellar DNA inheritance and predicted paternal and maternal inheritance patterns, but the reliability of the model must be experimentally verified. Specifically, model accuracy may be evaluated by experimentally demonstrating organellar DNA inheritance and comparing actual genetic data with the results predicted by the model. Accordingly, the degree of agreement between the theoretical model and the actual inheritance mechanism can be confirmed, which will enable an evaluation of model validity. We emphasize that our analyses identify statistical associations rather than causal mechanisms, and therefore the interpretations presented here should be viewed as hypotheses that merit further experimental investigation, particularly those testing the environmental effects on plastid transmission.

## CONCLUSIONS

We created a classification model for inheritance modes using trait data obtained from a database and plastid DNA inheritance modes determined by previous studies. Several traits showed statistical associations with inheritance patterns, although the strength and robustness of these associations varied among traits. Additionally, trait tendencies in seed plants were similar to those in conifers, in which paternal inheritance predominates, suggesting that similar ecological factors may be associated with plastid inheritance patterns across these groups. Although there were some limitations to these analyses, the accumulation of data regarding organellar DNA inheritance patterns may lead to the development of more sophisticated models and a better understanding of the evolutionary background of genetic patterns and the influence of environmental factors on inheritance.

## AUTHOR CONTRIBUTIONS

T.K. and T.M. designed the study. T.M. supervised the study. T.K. analyzed the data and wrote the manuscript. T.M. revised the manuscript. T.K. and T.M. approved the final manuscript.

## Supporting information


**Appendix S1:** The 378 species used for phyloglm models in this study and their plastid inheritance patterns.

**Appendix S2:** The number of individuals used to calculate the mean for each species and each trait.

**Appendix S3:** Plastid inheritance patterns, log10‐transformed and standardized trait values, imputation uncertainty, and observed/imputed status for the 378 species used in the analysis.

**Appendix S4**: Numbers and proportions of empirically observed and BHPMF‐imputed values for each trait used in the phyloglm analyses.

**Appendix S5:** Best‐supported and runner‐up models from the leave‐one‐clade‐out (LOCO) analyses, with median AIC, Akaike weights, and ΔAIC.

## Data Availability

Analysis scripts for the simulations and statistical analyses are available on GitHub (https://github.com/T-KONUMA55/cp_pattern).
